# Peripartum Cardiomyopathy: Current Insights into Pathogenesis and Clinical Management: A Narrative Review

**DOI:** 10.3390/jcm15082974

**Published:** 2026-04-14

**Authors:** Marzena Laskowska

**Affiliations:** Department of Obstetrics and Perinatology, Medical University of Lublin, Al. Racławickie 1, 20-069 Lublin, Poland; melaskowska@go2.pl or marzena.laskowska@umlub.edu.pl

**Keywords:** pregnancy complication, peripartum cardiomyopathy, etiopathogenesis, pregnancy and postpartum management, risk factors

## Abstract

Peripartum cardiomyopathy (PPCM) is a distinct condition that presents as heart failure (HF) in a woman who was previously healthy and has no prior cardiovascular issues. It results from idiopathic left ventricular (LV) dysfunction, characterized by a reduced LV ejection fraction below 45%. PPCM is a life-threatening condition with a high mortality rate (MR) that demands urgent treatment. Methods: This narrative review aims to define PPCM and its pathophysiology and conduct a scoping review of the latest data on the management of patients with peripartum cardiomyopathy during pregnancy and the postpartum period. Results: Currently, treatment follows standard HF protocols for reduced ejection fraction, with the possible addition of bromocriptine, and during pregnancy, medications that do not harm the fetus. Conclusions: Early, aggressive therapy is essential for a better prognosis, but managing PPCM can be challenging. Treatment of PPCM patients should be led by a team of highly qualified specialists, known as the Obstetric and Cardiac Care Team, comprising an obstetrician-perinatologist, an anesthesiologist, a cardiologist, and a cardiac intensive care specialist. Baseline left ventricular end-diastolic diameter (LVEDD) and left ventricular ejection fraction (LVEF) are the main prognostic factors. LVEF less than 30%, significant LV dilatation, LVEDD ≥ 6.0 cm, and right ventricular involvement are factors indicative of a poor prognosis. While pregnancy after PPCM is possible, it should be discouraged due to the significant risk of complications and even death. The most common causes of death in patients with PPCM are thromboembolic complications, severe HF, serious ventricular arrhythmias, cardiogenic shock, and sudden cardiac arrest.

## 1. Introduction

Although limited data on the incidence and prevalence of cardiovascular disease (CVD) in pregnancy are available for most parts of the world, it is estimated that heart disease occurs in 1% to 4% of all pregnancies and is the most common non-obstetric cause of maternal death related to pregnancy, childbirth, and the postpartum period, even in developed countries [1,2,3].

Unfortunately, mortality from cardiovascular disease during pregnancy is steadily increasing. In developed countries, cardiovascular disease has become the most common cause of death during pregnancy and the postpartum period, significantly outpacing obstetrical causes of death, such as bleeding or thromboembolism. Heart disease-related issues in pregnant women pose a significant ongoing challenge not only for cardiologists but also for primary care physicians and obstetricians. It is important to note that only approximately 17% of deceased mothers had a prior history of cardiac problems, while the majority appeared healthy before pregnancy [1,2,3].

In the USA, 17% to 25% maternal deaths, and in the UK, approximately 20%, are due to cardiovascular causes. By far the most common cardiovascular disease during pregnancy is hypertension, accounting for approximately 5–10% [1,2,3,4]. The leading causes of cardiovascular death during pregnancy include peripartum cardiomyopathy, ischemia, arrhythmias, aortic dissection, and myocardial infarction. Despite numerous studies, peripartum cardiomyopathy (PPCM) remains a poorly understood and dangerous disease. It causes heart muscle damage and decreased contractility, leading to heart failure in previously healthy women at the end of pregnancy or up to six months after delivery. While symptoms of the disease may resemble common pregnancy ailments, such as increased fatigue, shortness of breath, or peripheral edema, they should not be underestimated, as peripartum cardiomyopathy can lead to catastrophic consequences.

PPCM is a life-threatening condition requiring urgent treatment.

The clinical course of the condition can be varied and very dynamic. It can lead to severe heart failure with rapid deterioration. The mortality rate for peripartum cardiomyopathy can reach up to 30%. The first symptoms of the condition or mild to moderate cases of PPCM are often confused with physiological changes associated with pregnancy, especially in the postpartum period, and can easily be missed. Treatment of PPCM patients should be led by a team of highly qualified specialists, also known as the Obstetric and Cardiac Care Team, consisting of an obstetrician-perinatologist, an anesthesiologist, a cardiologist, and a cardiac intensive care specialist. Every patient diagnosed with PPCM requires long-term treatment and monitoring due to the risk of recurrence of cardiomyopathy, occurrence of complications, arrhythmia, or sudden cardiac events.

Peripartum cardiomyopathy is a poorly understood and dangerous condition, an idiopathic, potentially fatal form of dilated cardiomyopathy that develops during the final month of pregnancy or within five months after delivery. PPCM continues to pose a serious threat to the health and life of pregnant women and mothers after delivery. Unfortunately, the incidence of PPCM is increasing, and the etiology of this specific form of heart failure remains a matter of speculation. To date, no early biomarker has been identified that predicts the development of PPCM. This has direct clinical implications. Treatment of PPCM presents unique challenges for clinicians due to its complex pathogenesis, difficult-to-diagnose onset, and unpredictable fetal and maternal risks. Symptoms may initially be limited to low cardiac output syndrome with varying degrees of severity, or, rarely, PPCM begins with decompensated heart failure and cardiovascular collapse. Bromocriptine and other experimental therapies used to treat PPCM, aimed at blocking pathogenic pathways, have yielded inconsistent results. Therefore, this review focuses on the latest and most important observations regarding the etiopathogenesis of PPCM, the controversies surrounding breastfeeding, and the role of prolactin in the development of PPCM. This narrative review also describes the limitations and challenges related to treatment, personalized treatment methods, and future prognosis. This narrative review aims to provide a comprehensive overview of the pathophysiological processes associated with peripartum cardiomyopathy, synthesizing the latest discoveries and theories in the diagnosis and modern treatment of PPCM, to ensure not only updated knowledge but also the ability to apply it in everyday patient care.

## 2. Materials and Methods

### 2.1. Search Strategy and Eligibility Criteria

A comprehensive search of only English-language articles published in peer-reviewed scientific journals was conducted in Scopus, PubMed, and Web of Science using a predefined set of keywords. These terms were selected using the Medical Subject Headings (MeSH) system. The final search string was: (“peripartum cardiomyopathy” OR “peripartum heart failure” OR “right ventricular dysfunction” OR “left ventricular dysfunction”) AND (“treatment” OR “management” or “pharmacologic interventions” OR “ARNI” OR “SGLT2 inhibitors” OR “bromocriptine” OR “dopamine agonist”, OR “breastfeeding”) AND (“pregnancy” OR “gestation” “prenatal” OR “postpartum period”). The search included titles, abstracts, and keywords.

The inclusion criteria for this review included original clinical science studies, clinical trials, randomized controlled trials, cross-sectional studies, case–control studies, or cohort studies that focused on the pathophysiology and treatment of PPCM, studies strictly related to PPCM, pregnancy, and heart failure. A manual search for additional relevant studies and review articles was also conducted using references from the retrieved articles. The results obtained from the selected articles with a focus on recent discoveries of clinical relevance were then analyzed and organized for presentation in the appropriate sections of this narrative review.

### 2.2. Study Selection

To minimize the risk of bias, the initial screening was conducted without disclosing the authors’ affiliations or the study results. Articles that appeared relevant based on their titles and abstracts were selected for further analysis. The initially selected articles were then read in full to ensure that they met all of the inclusion criteria. The titles and abstracts of the remaining articles were then reviewed to determine their relevance. Full-text articles were then assessed for eligibility.

### 2.3. Data Extraction and Analysis

Data extraction from the selected articles was performed using a form designed to capture essential study characteristics. This form included information such as the authors, year of publication, country, and study design, as well as more detailed data relating to the pathophysiology of PPCM, diagnostic and therapeutic methods, medications used, and the key findings and conclusions of each study.

The results obtained from the selected articles were analyzed and organized for presentation in the appropriate sections of this review. The most significant findings were highlighted, and connections between various approaches to understanding the pathophysiology of peripartum cardiomyopathy and potential therapeutic targets were identified.

The purpose of this narrative review is primarily to summarize current pathophysiological hypotheses, review contemporary therapeutic strategies, and discuss prognostic factors and future research directions in peripartum cardiomyopathy. This narrative review also describes the limitations and challenges related to treatment, personalized treatment methods, and future prognosis.

## 3. Results

### 3.1. Definition

Peripartum cardiomyopathy (PPCM) is a distinct disease entity manifesting as heart failure (HF) in a previously healthy woman with no previous CVD, secondary to idiopathic left ventricular (LV) dysfunction with a reduced LV ejection fraction below 45%, which may or may not be associated with left ventricular dilatation that develops toward the end of pregnancy or in the months following delivery in the absence of another identifiable cause of cardiomyopathy [5,6,7]. A diagnosis requires ruling out other causes or conditions that could lead to heart failure [5,6,7]. The diagnosis of PPCM always requires differentiation from other conditions occurring in pregnant or postpartum women associated with circulatory decompensation. For this reason, PPCM is usually seen as a diagnosis of exclusion. PPCM is a life-threatening disorder and requires urgent treatment.

PPCM can occur in the third trimester of pregnancy or in the first 5–6 months after delivery, miscarriage, or abortion [5,8]. Most cases of PPCM occur after delivery, with 45% occurring in the first week and 75% in the first month after delivery [6,7,8]. It is important to note that although most cases of PPCM are diagnosed shortly after delivery, delayed symptoms can also occur, leading to significant diagnostic challenges and complicating treatment [9,10].

The occurrence of dyspnea in the peripartum period always requires careful evaluation and appropriate monitoring, as delayed diagnosis of peripartum cardiomyopathy can lead to catastrophic consequences. Postpartum surveillance and a thorough assessment of cardiopulmonary symptoms are necessary for every patient, even in the absence of other risk factors, to prevent delays in diagnosis and treatment.

### 3.2. Epidemiology

The prevalence of peripartum cardiomyopathy appears to vary according to race, geographical location, and diagnostic criteria. The incidence of peripartum cardiomyopathy varies significantly geographically and is estimated at 1/100 to 1/20,000 deliveries [7,8,10]. The highest incidence was reported in Nigeria and Haiti (10/1000), the lowest in the United States (1-4/1000), and in Asian countries, in Japan (1/20,000 deliveries) [6,7]. PPCM is four times more common in African American women than in Caucasian women [10,11].

### 3.3. Risk Factors

#### 3.3.1. Demographic Risk Factors

Risk factors for PPCM, in addition to ethnicity and Black race, include multiparity, multiple pregnancies, family history, status post-infertility treatment, anemia, and malnutrition. PPCM is more common in young women under 20 years of age and in patients over 40 or even 30 years of age [12,13].

#### 3.3.2. Obstetric and Cardiovascular Risk Factors

Significant risk factors for the condition include a history of peripartum cardiomyopathy, hypertension, preeclampsia, obesity, and diabetes, and prolonged use of tocolytic beta-agonists. Women with PPCM and preeclampsia have been reported to have a higher risk of serious neonatal complications, as well as a higher probability of recovery of left ventricular function (LVEF ≥ 50%). Prematurity and low birth weight are more common in infants born to mothers with PPCM and preeclampsia. Additionally, a 3.4-fold higher incidence of CVD and a 5-fold higher mortality rate were observed in children born to preeclamptic mothers with PPCM. The presence of multiple risk factors significantly increases the likelihood of PPCM occurring.

A valuable source of knowledge on the management of cardiomyopathies is the Registry of Pregnancy and Cardiac Disease (ROPAC) [5,14,15] and the EUR Observational Research Program (EORP), as well as an initiative of the Working Group on Peripartum Cardiomyopathy of the Heart Failure Association together with the PeriPartum CardioMyopathy registry, the aim of which is to conduct a comparative analysis of the clinical data of patients with PPCM from many countries associated with the European Society of Cardiology (ESC), with reference to data obtained from non-ESC countries [6,14].

### 3.4. Etiopathogenesis

The exact etiology of PPCM remains unclear [16,17]. The most frequently discussed theory of PPCM development is the “multiple hits” theory, which posits that genetic and environmental factors converge on a common pathway through several pathophysiological mechanisms.

It is believed to be a complex process, and one proposed mechanism is an angiogenic imbalance in the heart during the perinatal period in patients with genetic susceptibility [18].

The current hormonal-vascular hypothesis of peripartum cardiomyopathy [19] posits that antiangiogenic prolactin (PRL), cleaved from standard PRL under conditions of unbalanced oxidative stress, is the main causative factor of the disease [16,20,21].

Antiangiogenic PRL has a molecular weight of 16 kDa. The activation of cathepsin D, a protease that degrades PRL into an angiogenic and proapoptotic 16-kDa subfragment, plays an important role in producing antiangiogenic prolactin with a molecular weight of 16 kDa. This antiangiogenic prolactin induces endothelial dysfunction and damage, which subsequently leads to heart failure (HF). Cathepsin D (CathD) is secreted under conditions of high reactive oxygen species (ROS) concentration in the heart. The activity of cathepsin D is regulated by intracellular pH, metabolic products, hormones, growth factors, and inhibitors. Increased cathepsin D activation results from oxidative stress (OS), decreased antioxidant mechanisms, and decreased transcription of STAT3 activity [12].

16-kDa PRL causes cardiomyocyte damage and apoptosis, acting through microRNA-146a secreted from endothelial cells following stimulation by nuclear factor kappa beta (NF-κβ) and activation by complexes of 16-kDa PRL with plasminogen activator inhibitor-1 (PAI-1) [12,16,21]. When taken up by cardiomyocytes, miR-146a leads to metabolic disturbances by reducing glucose transporter type 4 (GLUT4) expression and glucose uptake. In the endothelium, miR-146a inhibits cell proliferation and promotes apoptosis, leading to impaired angiogenesis. Independent of miR-146a, 16-kDa PRL causes endothelial dysfunction by inducing caspase-dependent apoptosis and reducing the synthesis of inducible nitric oxide synthase (iNOS), leading to reduced nitric oxide (NO) production [21].

Inflammation unbalanced OS and systemic or local increases in other antiangiogenic factors, such as soluble fms-like tyrosine kinase receptor 1 (sFlt-1), have also been implicated in the development of PPCM and both local and systemic vascular dysfunction [20]. High sFlt1 concentrations in the blood during late pregnancy neutralize vascular endothelial growth factor (VEGF) family proteins (including VEGFA, VEGFB, and PLGF). VEGFA is crucial for the health of the heart’s blood vessels, and VEGFB likely plays a role in the endothelial transport of fatty acids, which are the primary energy source for cardiomyocytes [20]. This leads to reduced PGC-1α-dependent VEGF secretion, and therefore a lower toxicity threshold for sFlt1. ROS, Cathepsin D, and 16-kDa PRL are increased [20]. Endothelial dysfunction induced by sFlt1, therefore, leads to cardiomyocyte ischemia, metabolic failure, and apoptosis. Oxytocin may also have vascular toxicity, but its definitive role in peripartum cardiomyopathy has not been directly established [21,22].

Many other peptide hormones secreted by the aging placenta in late pregnancy have also been suggested to play a role in the etiopathogenesis of PPCM. Activin A is likely to contribute directly to cardiomyocyte dysfunction [23]. Activin A levels have been observed to correlate with subclinical cardiac dysfunction [23]. The importance of progesterone, which promotes cardiac hypertrophy and may have a direct negative inotropic effect on the heart by sensitizing cardiomyocytes to damage, has also been suggested [19,24]. The role of reduced relaxin levels in the development of PPCM has also been suggested.

It is also believed that a maladaptive response to the hemodynamic stress of pregnancy may lead to PPCM [25]. An immunological mechanism, involving the sensitization of maternal antibodies to fetal cells, has been hypothesized. These antibodies could sensitize maternal antibodies to myocardial epitopes, explaining why some patients with PPCM have antibodies against cardiac myosin heavy chains [25]. Hilfiker et al. suggested that PRL metabolism may also play a role in impaired cardiomyocyte angiogenesis, leading to HF [20]. Lampert et al. found an association between prolonged tocolysis and PPCM [26].

Heterozygous loss-of-function genetic variants in one of several genes known to be associated with ischemic dilated cardiomyopathy, a disease that partially resembles peripartum cardiomyopathy, have also been observed in approximately 15% of women with PPCM [27,28]. Furthermore, the frequency of these variants in dilated and peripartum cardiomyopathies was nearly identical, suggesting the importance of genetic predisposition. The mechanism by which hormonal factors are associated with these conditions remains unclear.

### 3.5. Clinical Presentation

PPCM most often occurs in the postpartum period and is therefore also called postpartum cardiomyopathy. Symptoms of left ventricular dysfunction may appear at the end of pregnancy or in the antenatal period. The clinical picture is dominated by the typical symptoms of heart failure. Dyspnea, significant respiratory effort, orthopnea, chest tightness, palpitations, tachycardia, and peripheral edema are predominant. Physical examination reveals rales over the lung fields, jugular vein distension, and less frequently, cardiogenic shock, thromboembolic complications, severe cardiac arrhythmias, and/or sudden cardiac arrest [7].

Diagnosis can be difficult because the symptoms of PPCM can be subtle, sometimes masked by symptoms occurring in late pregnancy. Mild to moderate symptomatic cases of PPCM are often confused with physiological changes associated with pregnancy, especially in the postpartum period, which is why most of these patients initially consult a gynecologist.

Although most women with peripartum cardiomyopathy have moderate or even severe symptoms of heart failure in NYHA class III or even IV, sometimes few physical symptoms occur despite significant cardiac dysfunction, especially in the early stage of the disease [29].

The clinical course of the condition can be very dynamic, and peripartum cardiomyopathy can have a rapid or even catastrophic course, including cardiogenic shock [11]. Most women develop acute heart failure (AHF) with severe symptoms of New York Heart Association class III or IV HF, with rapid deterioration and high mortality ranging from 10% to 28%, half of which occurs within the first month after diagnosis [7].

The most common causes of death in patients with peripartum cardiomyopathy are thromboembolic complications, severe HF, and serious ventricular arrhythmias. In the least complicated cases, complete recovery and resolution of HF symptoms and LV dysfunction may occur.

### 3.6. Diagnosis

The gold standard for diagnosis is echocardiography (ECHO), as well as measuring the levels of the N-terminal fragment of the brain-type natriuretic peptide (NT-proBNP).

Diagnostic criteria for PPCM require a reduction in the left ventricular ejection fraction (LVEF) below 45%, with the exclusion of pre-existing heart disease and other causes of heart failure. Echocardiography also reveals left ventricular dilatation with global hypokinesia and pseudonormal diastolic filling [9]. Additionally, functional mitral regurgitation and right ventricular dysfunction may be present. Echocardiographic evaluation helps assess intracardiac thrombi, assess the severity of PPCM, and exclude congenital heart defects [30]. Although the assessment of the N-terminal fragment of the natriuretic peptide (NT-proBNP) is nonspecific, it is a highly sensitive indicator. Plasma levels of brain natriuretic peptide (BNP) do not typically change during a normal pregnancy and are usually elevated in patients with peripartum cardiomyopathy, which may prompt the consideration of this diagnosis [11].

Normal NP levels are not typically observed in patients with heart failure due to newly diagnosed peripartum cardiomyopathy [30].

Significant left ventricular dilatation with a left ventricular end-diastolic diameter (LVEDD) of ≥6.0 cm, a left ventricular ejection fraction of less than 30%, and right ventricular involvement are factors indicative of a poor prognosis [7,21,31].

Although there are no specific electrocardiogram (ECG) features of peripartum cardiomyopathy, the results are usually abnormal. The most common findings are left ventricular hypertrophy and overload; intraventricular conduction disturbances, most left bundle branch block; QT prolongation; and T-wave and ST-segment abnormalities, e.g., T wave inversion and ST segment depression [30,32,33,34].

Further diagnostic workup for PPCM includes genetic testing and magnetic resonance imaging (MRI). It is estimated that approximately 20% of patients with postpartum cardiomyopathy have mutations in genes encoding cardiomyocyte structural proteins, which are characteristic of both postpartum cardiomyopathy and dilated cardiomyopathy [11,35].

Currently, there is no specific diagnostic biomarker for peripartum cardiomyopathy.

Genetic testing is increasingly being offered to patients with peripartum cardiomyopathy and should be considered in most cases, especially in cases of familial cardiomyopathy [11]. Cardiac magnetic resonance imaging with late gadolinium enhancement is considered a sensitive and reproducible imaging technique for assessing myocardial fibrosis. Persistent cardiac dysfunction is attributed to focal myocardial damage. Cardiac MRI helps exclude other cardiomyopathies, although typical findings in PPCM are nonspecific, such as global dysfunction, myocardial edema, and subtle late gadolinium enhancement [9]. Although there is no specific pathognomonic cardiac MRI appearance in peripartum cardiomyopathy, this examination can be helpful in patients with suspected PPCM, not only for assessing right and left ventricular function and dimensions, but also for assessing the structure of the myocardium. Many patients with PPCM have MRI findings of regional wall contractility abnormalities, myocardial edema, focal non-ischemic intramyocardial or subepicardial late gadolinium enhancement, usually anteroseptal, nonspecific pericardial effusion, and occasionally left ventricular thrombi. Left ventricular dilatation is often accompanied by hypertrabeculation [30].

Endomyocardial biopsy is not recommended for the diagnosis of postpartum cardiomyopathy due to its high risk during pregnancy and low sensitivity, coupled with the lack of specific features of this form of cardiomyopathy on histopathological examination. Endomyocardial biopsy may help detect other causes of AHF, such as lymphocytic, eosinophilic necrotizing, and giant cell myocarditis.

### 3.7. Peripartum Cardiomyopathy—Differential Diagnosis

The differential diagnosis of PPCM should include myocardial infarction, pulmonary artery embolism, previously unrecognized congenital heart disease, previously unrecognized valvular heart disease, hypertension, preeclampsia, and pre-existing idiopathic or familial dilated cardiomyopathy revealed during pregnancy due to circulatory system stress.

It is necessary to exclude myocardial infarction (MI), aortic dissection, viral myocarditis, and renal artery stenosis. Tests include serum troponin and brain NP levels, renal function assessment, and ECG and ECHO [25]. Infiltrative heart disease can be diagnosed with an MRI. Furthermore, angiography may be necessary to detect myocardial infarction (MI). The differential diagnosis of myocarditis is based on the evaluation of clinical symptoms and laboratory and imaging symptoms. The symptoms are often nonspecific and mimic other cardiac or systemic conditions. Common symptoms include chest pain, palpitations or arrhythmia, dyspnea at rest or with exertion, chronic fatigue, weakness, fever, muscle and joint pain, fainting or sudden loss of consciousness, and swelling of the lower limbs. An electrocardiogram may reveal nonspecific changes characteristic of myocardial damage or arrhythmias.

Laboratory tests may reveal elevated troponin levels, which indicate cardiomyocyte damage. They may also reveal elevated inflammatory markers, such as C-reactive protein (CRP) and leukocytosis, as well as elevated levels of N-terminal pro-brain natriuretic peptide (NT-proBNP) or brain natriuretic peptide (BNP), which indicate heart failure. In selected cases, serological and immunological tests may be helpful. Echocardiography and cardiac magnetic resonance imaging may reveal areas of inflammation and muscle damage, signs of heart failure, or the presence of pericardial effusion. Endomyocardial biopsy may help make the diagnosis.

### 3.8. Peripartum Cardiomyopathy—Treatment

The treatment of PPCM remains a significant challenge. Due to the high risk of cardiopulmonary failure, the need for continuous hemodynamic monitoring, and the necessity of access to mechanical ventilation and circulatory support, it should be conducted in intensive care units (ICUs). Prompt diagnosis and the formation of an obstetric cardiothoracic team, i.e., a multidisciplinary team consisting of cardiologists, obstetricians-maternal–fetal medicine specialists, anesthesiologists, neonatologists, intensive care specialists, and possibly cardiac surgeons, and the implementation of an appropriate treatment algorithm are crucial for successful therapy and prognosis [36].

Treatment of peripartum cardiomyopathy depends on the timing of disease onset.

Therapeutic interventions must always consider the health of the mother and fetus, considering the adverse effects of medications on the fetus and the fetoplacental circulation.

Postpartum therapy generally follows the European Society of Cardiology (ESC) guidelines for the standard treatment of heart failure with reduced ejection fraction, with particular attention paid to the risk of thromboembolic complications and the risk of severe, life-threatening cardiac arrhythmias in the postpartum period.

Patients with severe HF will require invasive monitoring, including the measurement of blood pressure, central venous pressure, and pulmonary artery catheterization. Patients in shock require inotropic and vasopressor therapy. If they are refractory to pharmacological treatment, they may require short-term support devices such as extracorporeal membrane oxygenation, or long-term devices such as a left ventricular assist device (LVAD).

In emergencies, both cardioversion and electrical defibrillation are therapeutic options for pregnant women of any gestational age and postpartum in any patient with medical indications for such treatment [6,36,37]. In these clinical situations, fetal monitoring should always be implemented to exclude secondary fetal arrhythmias [7,37,38].

The treatment of PPCM should include bromocriptine, heart failure therapy, anticoagulation, and vasodilators. In selected cases, diuretics may also be used. Non-invasive ventilation should be used for patients with pulmonary congestion [16].

The European Society of Cardiology (ESC) recommends adding bromocriptine to standard heart failure therapy. The initial dose is 2.5 mg twice daily for two weeks, followed by 2.5 mg once daily for six weeks [6,7,8,20,35,36,38,39].

In selected cases, a slightly different dosing regimen may be used: 2.5 mg of bromocriptine for 7 days, followed by 5 mg/day for 2 weeks, then 2.5 mg daily for the next 6 weeks.

Bromocriptine reduces concentrations of prolactin and its deleterious derivatives in peripartum cardiomyopathy and is considered a PPCM-specific causal therapy [39].

Significant improvement in left ventricular systolic function and regeneration and efficacy in preventing heart failure in subsequent pregnancies has been reported in women with PPCM treated with bromocriptine in a multinational study conducted by the European Society of Cardiology (ESC) EURO observational Research Program on PPCM [6,7,8,20,30,35,36,38,39]. It is recommended that the decision to introduce bromocriptine be considered on an individual basis for each patient. Improvement of left ventricular regeneration has also been observed in lactation suppression alone. In the absence of bromocriptine, cabergoline can be used for the same purpose at a dose of 0.5 mg or 1 mg per week [40].

The Randomized Evaluation of Bromocriptine in Myocardial Recovery Therapy for Peripartum Cardiomyopathy (REBIRTH) study, currently underway in the USA, aims to evaluate the effectiveness of bromocriptine in myocardial recovery therapy for patients with peripartum cardiomyopathy and assess the adverse effects of this therapy. The results of these studies are expected in 2029 [30,34].

The hypercoagulable state characteristic of pregnancy is an additional cause of increased risk of thrombotic complications, including left ventricular thrombosis and thromboembolic events, observed in 5–20% of cases [41]. Patients with PPCM require anticoagulation for up to 6 weeks postpartum due to the high risk of thromboembolic complications, particularly in women treated with bromocriptine [41]. Patients with embolism, cardiac thrombus, severe dilatation, and LV dysfunction should receive anticoagulation until LV function normalizes. The drug of choice for the prevention and treatment of thromboembolic complications in pregnant women with PPCM is unfractionated or, more commonly, low-molecular-weight heparin [7,8].

The standard indication for initiating heparin therapy is an LVEF <35% and/or bromocriptine therapy or the presence of atrial fibrillation (AF) [7,8,41].

Digitalis preparations can be used during pregnancy and lactation but serum drug concentrations should always be closely monitored due to the possibility of overdose, especially when used in combination with diuretics. Use of this drug in breastfeeding women requires special caution because digoxin passes into breast milk and there is a risk of exposure to drugs in neonates during the lactation period due to maternal drug intake. However, digoxin passes into breast milk in minimal amounts, and the drug concentration in breast milk is too low to significantly affect the newborn. Therefore, breastfeeding is not absolutely contraindicated when used in low therapeutic doses when there are absolute indications [42]. Improvement in clinical condition and a simultaneous reduction in the risk of relapse have been reported with digoxin therapy for 6 to 12 months.

β-Blockers should also be considered in the therapy of PPCM [43]. They can be used both during pregnancy and postpartum because they improve clinical condition, LVEF, and survival in PPCM.

Cardioselective agents from this group are preferred during pregnancy [44].

Sartans (angiotensin II receptor blockers—ARBs) and angiotensin-converting enzyme inhibitors (ACEIs) are contraindicated during pregnancy due to the risk of malformations, and negative effects on placental perfusion and the fetal renal system, resulting in renal failure and oligohydramnios [8,30,44]. Similarly, diuretics (hydrochlorothiazide, furosemide) are contraindicated during pregnancy because they pose a risk of reduced placental perfusion [44]. Diuretics should only be used in specific situations, such as pulmonary congestion, volume overload, and pulmonary edema. In such cases, furosemide can be used at an initial dose of 20–40 mg [44]. Outside of pregnancy and lactation, diuretics can be used without these restrictions. These drugs improve the patient’s clinical condition. Aldosterone antagonists (spironolactone) alleviate symptoms, reduce the frequency of hospitalizations in patients with severe HF (NYHA—New York Heart Association) class III and I, and reduce mortality, but can only be used outside of pregnancy.

Rapid and aggressive oxygen therapy is also crucial, aiming to achieve saturation >95%. In some cases, non-invasive ventilation with positive end-expiratory pressure of 5–7 cm H_2_O is necessary. Hydralazine is a safe drug during pregnancy. It dilates blood vessels, reduces vascular resistance and afterload, and lowers blood pressure. Hydralazine improves maternal and fetal circulation, which significantly contributes to organ protection [43].

In emergencies, nitroglycerin can be used intravenously at a dose of 10 to 20 µg/min, up to a maximum of 200 µg/min, for the same purpose [41]. Remember not to use nitroglycerin in patients with systolic blood pressure ≤100 mmHg due to the risk of hypotension [7,8,45]. For patients diagnosed with peripartum cardiomyopathy who exhibit symptoms of low cardiac output and congestion who have not responded to vasodilators and diuretics, levosimedan is recommended as the primary inotropic drug at a dosage of 0.1 µg/kg/min for 24 h. Levosimendan improves hemodynamic function without activating the sympathetic nervous system and increases the force of myocardial contraction without impairing ventricular relaxation. Levosimendan opens ATP-dependent potassium channels in vascular smooth muscle, causing the dilation of arterial resistance vessels (including coronary vessels) and venous capacitance vessels. After a trial with levosimedan, dobutamine may be used as a second-line inotropic drug. Epinephrine may be used in this group of patients exceptionally during resuscitation [6,46,47].

The use of pentoxifylline in patients with PPCM, combined with standard therapy, leads to improved cardiac function, as measured by the NYHA classification, increased LVEF (left ventricular ejection fraction), and reduced mortality [6].

Alternative therapies, including monoclonal antibodies, interferon, immunosuppression, and immunoglobulins, have also been mentioned in the treatment of PPCM.

Newer medications for heart failure due to dilated cardiomyopathy with reduced ejection fraction, such as sacubitril + valsartan, an angiotensin receptor-neprilysin inhibitor (ARNIs), and sodium-glucose cotransporter 2 (SGLT2) inhibitors, are sometimes used in patients with peripartum cardiomyopathy.

In the PARADIGM-HF study, sacubitril/valsartan, compared with enalapril, reduced the risk of hospitalization for heart failure, cardiovascular death, and all-cause mortality to a similar extent in women and men with heart failure with reduced ejection fraction. Furthermore, sacubitril/valsartan was safe and well-tolerated, regardless of gender. It should be noted that there is insufficient data on the safety and use of these medications during pregnancy and the postpartum period, and they should be avoided during pregnancy [9,11]. Sacubitril and valsartan work by inhibiting the enzyme neprilysin and blocking the angiotensin II receptor. Sodium-glucose cotransporter-2 (SGLT2) inhibitors, such as dapagliflozin and empagliflozin, act in the kidneys to increase glucose excretion in urine and help remove excess sodium and fluid. They work by blocking the protein that transports glucose and sodium in the kidneys (SGLT2), which lowers blood sugar levels and leads to calorie loss, and therefore weight loss. These drugs also have positive effects on the cardiovascular system and kidneys and do not cause hypoglycemia [9,11]. Evidence supporting the effectiveness of this therapy for PPCM is limited.

Modern medications used in the treatment of heart failure with reduced ejection fraction (HFrEF), such as ARNI (sacubitril/valsartan, an angiotensin receptor and neprilysin inhibitor) and flozins–SGLT2 (sodium-glucose cotransporter type 2) inhibitors, have been proven to be highly effective. A reduction in cardiovascular mortality and the risk of sudden cardiac death associated with harmful ventricular arrhythmias has been reported in patients with HFrEF treated with angiotensin receptor neprilysin inhibitors (ARNIs).

The mechanism of this effect is not entirely clear [48]. The latest drugs from the flozins group have special properties. Studies have shown that SGLT2 inhibitors have beneficial cardioprotective effects. These effects include protecting the antioxidant system at the cellular level, improving cardiac cell metabolism and endothelial function, and slowing down myocardial fibrosis. For this reason, SGLT2 inhibitors are considered an integral part of heart failure treatment [49].

Clinical trials have shown that ARNI and SGLT2 combined use provides additional benefits in patients with heart failure, especially in patients with reduced ejection fraction, as these drugs’ mechanisms of action are complementary; therefore, combined treatment appears to lead to a greater reduction in the occurrence of cardiovascular events and serious complications, as well as to reduce mortality [9].

Although ARNI and SGLT2 inhibitors are effective in the treatment of heart failure with reduced ejection fraction (HFrEF), they are used off-label in patients with PPCM and require extreme caution and individual patient consent for off-label treatment [9,11,42,50,51].

All available medications for HF can be used in patients with PPCM after delivery, including β-blockers, ACEIs, angiotensin receptor inhibitors (ARNIs), mineralocorticoid receptor antagonists, and SGLT2 inhibitors, as well as digoxin in patients with sinus tachycardia or atrial fibrillation with a rapid ventricular rate in postpartum patients who are not breastfeeding [30].

Although beneficial results have been observed with sacubitril/valsartan, an angiotensin receptor neprilysin inhibitor (ARNI), in the treatment of HFrEF, its usefulness in peripartum cardiomyopathy is less documented. It appears that the use of sacubitril/valsartan therapy in selected patients who are intolerant to conventional ARNI therapy may be a valuable alternative in facilitating cardiac recovery [50,51].

Special caution should be exercised when using standard medications for the treatment of heart failure in patients with PPCM during lactation [30].

European Society of Cardiology guidelines suggest suppressing lactation. Some believe that breastfeeding is sometimes possible, especially in milder forms of PPCM. To date, breastfeeding remains controversial in patients with PPCM [30].

According to the National Institutes of Health Drugs and Lactation Database in the USA, metoprolol (a beta-blocker), ACEIs, spironolactone (a mineralocorticoid receptor antagonist), and ARNIs can be used with extreme caution. It should be emphasized that data on the safety of this drug for infants are very limited [30]. SGLT2 inhibitors should be avoided [30].

For patients with severe heart failure during PPCM, both during pregnancy and the postpartum period, extracorporeal membrane oxygenation (ECMO) is an available therapeutic option [52]. The indications for ECMO in patients with PPCM are the same as those for extremely severe circulatory failure [52,53].

If conservative methods are ineffective, heart transplantation will be the last therapeutic option [36]. It is estimated that as many as 10% of patients with PPCM will require heart transplantation, and the median time from the onset of PPCM to heart transplantation is approximately 140 days [54]. In the USA, as many as 5% of heart transplants are performed in patients with peripartum cardiomyopathy [54]. Furthermore, data indicate a poorer prognosis in women undergoing heart transplantation due to PPCM than in women undergoing transplantation for other medical indications. Unfortunately, the treatment of HF in women with PPCM and persistent left ventricular dysfunction is a long-term therapeutic procedure. It is recommended that ACEI and β-blocker therapy be continued for no less than 12 months after full recovery of left ventricular systolic function and normalization of left ventricular ejection fraction (LVEF).

For many patients with a history of PPCM and normal LVEF, no recurrence of the disease was observed for several years after treatment was discontinued, clinical improvement was achieved, and echocardiogram parameters were normalized. Nevertheless, it should be emphasized that many women in this group demonstrated a lesser ability of the left ventricle to adapt to hemodynamic stress, as well as a lower left ventricular contractile reserve. This suggests the presence of persistent subclinical left ventricular dysfunction in women with a history of PPCM.

If pharmacotherapy is ineffective, circulatory support may be necessary using an intra-aortic balloon pump (IABP) or an implantable left ventricular assist device (LVAD) [44]. This therapy can also serve as a bridge to qualifying for and awaiting a heart transplant [52,53].

Persistent dysfunction and symptoms of heart failure after 6 months of diagnosis, despite optimal therapy, constitute an indication for implantable cardioverter-defibrillator (ICD) implantation for primary prevention in patients with NYHA functional class II and III and LVEF <35% [39] if the simultaneous coexistence of QRS widening >120 ms in patients with NYHA functional class II to IV constitutes an indication for implantation of a cardiac resynchronization therapy defibrillator (CRT-D) pacemaker [39]. Implantation of an implantable cardioverter-defibrillator before therapy is usually not recommended, as the heart rate typically returns to normal within six months of therapy in most patients with LVEF ≥35% [7]. It should be noted that PPCM also carries an increased risk of serious ventricular arrhythmias.

The AHA and ESC recommend that in patients with PPCM and persistent cardiac arrhythmias, the temporary use of wearable cardioverter-defibrillator (WCD) devices be considered to prevent sudden cardiac death with LVEF ≤35% [6,8,13].

### 3.9. Peripartum Cardiomyopathy—Time and Mode of Delivery

The management and care of a pregnant woman with peripartum cardiomyopathy requires close collaboration among the entire obstetric cardiology team, which includes a cardiologist, an anesthesiologist, an intensivist, a perinatologist, a neonatologist, and sometimes a cardiac surgeon.

In stable patients, vaginal delivery with epidural anesthesia may be preferred in selected cases [32]. In severely ill patients, cesarean section is recommended [6,7,8]. Vaginal delivery provides greater hemodynamic stability, is associated with less blood loss, and has a lower risk of infectious and pulmonary complications. Labor analgesia reduces the adverse effects of pain. Regional anesthesia is preferred. Sympathetic blockades reduce cardiac preload and afterload, which is a significant factor in improving myocardial performance.

Patients with PPCM are at high perioperative risk; therefore, a multidisciplinary team approach is necessary, and anesthesia for cesarean section poses a significant challenge in this group of patients. Regardless of the route of delivery, special care is always necessary to maintain hemodynamic stability and avoid fluid overload [30].

Cesarean section carries a higher risk of hemorrhage and blood loss, as well as an increased risk of pulmonary edema. Epidural anesthesia with circulatory monitoring is preferred for cesarean delivery because it provides greater hemodynamic stability with a slow, progressive, primarily sensory block. Spinal anesthesia is not recommended due to the sudden hemodynamic changes associated with a single anesthetic administration, which increases the risk of pulmonary edema and sudden cardiac arrest. Regional anesthesia is usually preferred over general anesthesia. Sometimes general anesthesia may be necessary. Anticoagulation therapy is a contraindication to regional anesthesia. In such cases, general anesthesia would be indicated. It should be noted that general anesthesia is associated with greater cardiovascular burden and an increased risk of bradycardia, left ventricular failure, pulmonary edema, and even sudden cardiac arrest.

### 3.10. Lactation and Breastfeeding

Medications used in PPCM can be used during lactation; however, most physicians and the recommendations of the European Society of Cardiology Working Group do not recommend breastfeeding in patients with PPCM due to the postulated association between the etiopathogenesis of peripartum cardiomyopathy and prolactin [3,7].

It appears that the inhibition of prolactin secretion, both through pharmacological agents and by simply discontinuing breastfeeding, may be beneficial in patients with peripartum cardiomyopathy [13]. The European Society of Cardiology guidelines recommend bromocriptine, but experts in the United States have not reached a consensus [3,11,13].

Only a few studies suggest that breastfeeding may be safe in women with peripartum cardiomyopathy [11,55,56,57]. Although there are potential benefits to using bromocriptine to treat PPCM, evidence of its effectiveness is limited and based on relatively small studies. Larger randomized studies are ongoing in both Europe and the USA. It has been observed that suppression of prolactin release using bromocriptine has been associated with more favorable outcomes, but it was a small study. Further clinical evaluation and more research is needed before therapy with bromocriptine.

There is still no consensus on whether bromocriptine should be used to treat patients with PPCM. Data regarding the use of bromocriptine to treat PPCM patients with cardiogenic shock are scarce. Bromocriptine is rarely used in the USA, though it is widely recommended in Europe [46]. To date, there are no completed randomized, placebo-controlled clinical trials investigating the effectiveness of bromocriptine in patients with PPCM. A poorer prognosis has been observed in patients treated with ECMO who had elevated prolactin levels [46]. Carbegoline also appears feasible when bromocriptine is unavailable [46].

Ongoing studies in Europe and the USA will provide greater insight into the role of bromocriptine in the treatment of PPCM.

### 3.11. Considerations for Breastfeeding in Patients with Postpartum Cardiomyopathy (PPCM)

While maternal treatment is the primary concern and breastfeeding is a secondary issue, it should be noted that evidence supporting the effectiveness of lactation suppression and the addition of bromocriptine to standard heart failure therapy in PPCM is limited and inconclusive. This evidence comes from a small number of studies with many confounding variables. Bromocriptine therapy is just one possible treatment. While almost all drugs pass into breast milk, the amount is usually low. Therefore, there is no clear justification for routinely using bromocriptine and completely excluding breastfeeding, even if the mother is taking medication for heart failure. Breastfeeding has many benefits for both mother and newborn. Breast milk is the best source of nutrition for infants and newborns, providing immunoprotective effects and reducing the risk of cot death and obesity. It also contributes to better psycho-cognitive development in newborns. Furthermore, breastfeeding women have been shown to have a lower risk of breast cancer, ovarian cancer, hypertension, and diabetes. Breastfeeding also promotes a positive relationship and bond between mother and child. While each decision should be made on an individual basis, it appears that in many cases, medication can be continued during breastfeeding.

Although sacubitril/valsartan is effective, it is not recommended for breastfeeding women due to concerns about its potential adverse effects on infant development. No previous studies have evaluated the transfer of NEP inhibitors (e.g., sacubitril) into human milk. Admittedly, a single case series has reported limited transfer of these drugs into human milk, but this lack of data restricts the use of these medications for breastfeeding mothers with heart failure (HF).

Data from literature are presented in Table 1.

### 3.12. Prognosis

Although peripartum cardiomyopathy is associated with significant morbidity and mortality, approximately 50% to 80% of patients recover normal left ventricular systolic function, with an ejection fraction above 50%, within six months of symptom onset [7,30,58,59].

The latest European data from ECS 2025 indicates a range of 25% to 62% of patients, depending on the geographic region. Partial recovery is defined as an improvement in left ventricular ejection fraction of at least 10% [61]. Lack of improvement after 6 or 12 months in most cases indicates irreversible myocardial damage [7].

Although cardiac function typically returns to normal in more than 50% of patients with peripartum cardiomyopathy, morbidity and mortality are still high, with some patients requiring implantation of a left ventricular assistance device (LVAD) or heart transplantation. Black women in the United States are twice as likely to experience permanent cardiac dysfunction as White women, and among Black women whose heart function returns to normal, it takes twice as long to do so [35]. Mortality rates are as high as 20% and are highest among Black women in the United States and among women in underdeveloped countries worldwide [11,15].

The main prognostic factors are the baseline dimensions of the left ventricle (LV EDD-LV end-diastolic diameter) and the left ventricular ejection fraction (LVEF). A positive correlation has been reported between a higher left ventricular ejection fraction and a favorable prognosis and recovery [37,38]. Some patients will require mechanical cardiac support or even heart transplantation [7,30,38,45,62].

A 21% risk of developing heart failure in a subsequent pregnancy has been observed in patients whose left ventricular ejection fraction (LVEF) has normalized. In patients with persistent systolic dysfunction, this risk is more than twice as high.

In patients with LV EDD >6.0 cm (LV end-diastolic diameter) and LVEF <30% (LV ejection fraction), no recovery of left ventricular function was observed in any patient. This group of patients is at higher risk of requiring mechanical support and heart transplantation. Most patients with LVEF >30% and LV EDD <6.0 cm usually experience improvement [37,38]. An additional independent prognostic factor is right ventricular function at the time of diagnosis of peripartum cardiomyopathy [63,64]. If it is normal, there is a greater chance of regeneration and recovery.

African American and Haitian patients have been reported to have a more severe course of PPCM, a more frequent reduction in left ventricular ejection fraction (LVEF) to <30% at diagnosis, and clinical deterioration despite the same treatment regimen in this group of patients [7,8]. Studies have been inconclusive, but a higher risk of heart transplantation and death has also been reported in Black women [7,8].

Other indicators of adverse outcomes include delayed medical consultation (more than 1 week postpartum), late gadolinium enhancement on MRI, and left ventricular dilatation [11,65].

The occurrence of preeclampsia with peripartum cardiomyopathy is associated with better left ventricular recovery, which may be due to the often-better baseline cardiac function and the reversible nature of preeclampsia. Despite these findings, no significant difference in mortality was found after 12 months [66].

Most life-threatening complications can be avoided only through early and aggressive treatment according to the guidelines. The lowest recovery rates were observed in women with delayed diagnosis, particularly those with clinical symptoms for more than 10 days before diagnosis and initiation of targeted treatment, a QRS duration of 110 ms or longer, and no preeclampsia [30].

Data from the ESC peripartum cardiomyopathy global registry showed that in countries with low healthcare spending, guideline-consistent heart failure therapy was less frequently recommended, and mortality within 6 months of diagnosis was higher [30,33,34]. Furthermore, increased rates of left ventricular dilatation and decreased rates of left ventricular recovery were observed among patients from countries with low healthcare spending and low education levels. Maternal outcomes, such as persistent left ventricular dysfunction, rehospitalization, or death, were also associated with income, and neonatal mortality was higher in countries with low healthcare spending but was not related to maternal education or income.

Considering these data, it is important to emphasize not only the importance of spending appropriate funds on healthcare and education, but also the importance of early diagnosis and the full treatment of peripartum cardiomyopathy in accordance with the guidelines to reduce complications and maternal and neonatal mortality [30,33,34].

It has also been suggested that peripartum cardiomyopathy may also be the first symptom of rare diseases such as Danon disease or Duchenne muscular dystrophy [28].

### 3.13. Complications

The most common complication is thromboembolic and is observed in 5–6% of cases. It can affect both the right and left heart chambers [5,38,39]. Risk factors for thromboembolic complications include cardiac dilatation, decreased contractility, and blood stasis, as well as endothelial damage [29,38,39]. It should be noted that pregnancy itself is a hypercoagulable state secondary to increased levels of coagulation factors VII, VIII, X, fibrinogen, and von Willebrand factor, and decreased activity of proteins S and C, and decreased fibrinolysis. These changes persist for 6 to 8 weeks postpartum [38,39,61,67].

Complications of PPCM also include severe cardiac arrhythmia, heart failure, and sudden cardiac death [68]. Approximately 50% of deaths occur within the first month after diagnosis of peripartum cardiomyopathy [67]. Prompt diagnosis and aggressive immediate treatment are crucial to reducing mortality and the risk of complications.

### 3.14. Pregnancy After Peripartum Cardiomyopathy

Pregnancy after PPCM is possible, but both the AHA and the ECS believe that subsequent pregnancy should be contraindicated in patients whose left ventricular ejection fraction (LVEF) has not achieved a normal value (no increase above 50–55%) due to the significant risk of complications [6,7,61,68]. Left ventricular ejection fraction (LVEF) is the strongest predictor of complications.

The decision to have another pregnancy after PPCM should be individualized, and the patient should be aware that a subsequent pregnancy may be associated with disease recurrence and the risk of death due to the burden on the circulatory system. Subsequent pregnancy places additional strain on the heart. Cardiological evaluation and echocardiography are necessary in each patient before a planned pregnancy to identify patients at higher risk of recurrence and patients with subclinical left ventricular dysfunction.

Although pregnancy can be successful in patients after intensive PPCM therapy with normalized LVEF treated postpartum with bromocriptine, there is a risk of recurrence and sudden cardiac death in a subsequent pregnancy, even if LVEF normalizes.

Even among patients who began pregnancy with a normalized left ventricular ejection fraction, a 20% risk of worsening left ventricular function was observed.

Women with a history of PPCM with a confirmed left ventricular dysfunction and a left ventricular ejection fraction (LVEF) <50% had a 50% risk of developing acute heart failure with progressively worsening cardiomyopathy in a subsequent pregnancy, and the risk of death in this group of patients was 25% to 50% [37,38].

Persistent left ventricular dysfunction also carries a higher risk of miscarriage, intrauterine fetal death, and preterm birth [37,38,68]. Close cardiological supervision is always necessary, along with the discontinuation of teratogenic medications used to treat heart failure 3 months before a planned pregnancy. In addition to close cardiologic monitoring, an echocardiogram with assessment of cardiac systolic function should be performed after discontinuation of the pharmacological treatment.

The occurrence of cardiac arrhythmias increases the risk of morbidity and mortality in PPCM. It is important to emphasize that sudden death caused by ventricular arrhythmias accounts for 25% of deaths in PPCM [37,38,68]. Even with normalized ventricular function, subsequent pregnancy carries the risk of recurrence of cardiomyopathy, heart failure, and permanent heart dysfunction [7,68]. For these reasons, prophylactic use of beta-blockers should be considered during subsequent pregnancies, even in patients with restored left ventricular systolic function. Systematic monitoring during pregnancy and for 6 months postpartum is recommended, including regular echocardiograms, clinical examination, and cardiologist evaluation. Beta-blocker therapy should not be discontinued when planning a pregnancy after PPCM. Reinitiating therapy with a beta-blocker may also be beneficial in subsequent pregnancies, regardless of baseline left ventricular systolic function.

### 3.15. Effective and Safe Contraception for Women After PPCM

Effective contraception should be discussed with each patient with PPCM by the obstetrician and cardiologist. Avoidance of estrogen-containing contraceptives is recommended, especially in the early postpartum period, due to the increased risk of thromboembolic complications associated with cardiac systolic dysfunction. Progestogen-releasing subdermal implants or progestogen-containing intrauterine devices are recommended as safe contraceptive methods in this group of patients. While pregnancy after PPCM is possible, it should be discouraged due to the significant risk of complications and even death. The most common causes of death in patients with peripartum cardiomyopathy are thromboembolic complications, severe heart failure, serious ventricular arrhythmias, cardiogenic shock, and sudden cardiac arrest.

## 4. Discussion

### 4.1. Main Findings

Peripartum cardiomyopathy is a potentially life-threatening condition, defined as heart failure (HF) with a reduced left ventricular ejection fraction (LVEF) <45%, without any other cause of heart failure, occurring primarily during the perinatal period or in the months following delivery. Peripartum cardiomyopathy is generally diagnosed after other causes of heart failure have been excluded, and its treatment remains a significant challenge. Prompt diagnosis and implementation of an appropriate treatment algorithm are crucial. PPCM during pregnancy requires joint care by cardiologists and obstetricians–an obstetric cardiology team, i.e., a multidisciplinary team.

The precise pathophysiological mechanisms underlying PPCM remain unclear, although the condition is widely considered multifactorial. This narrative review addresses not only the complex and multifactorial pathogenesis of peripartum cardiomyopathy but also practical aspects, focusing on two clinical goals: treatment and safe delivery. Treatment of heart failure in women with PPCM and persistent left ventricular dysfunction is a long-term treatment. It is recommended that therapy last no less than 12 months after the full recovery of left ventricular systolic function and normalization of LVEF.

In many patients with a history of PPCM and normal LVEF, no recurrence of the disease was observed for several years after clinical improvement, normalization of the echocardiogram parameters, and treatment discontinuation. Nevertheless, many women in this group experienced less adaptation of the left ventricle to hemodynamic stress and a lower left ventricular contractile reserve, suggesting the presence of persistent subclinical left ventricular dysfunction in women with a history of PPCM.

The use of bromocriptine in combination with standard heart failure therapy remains controversial. Although numerous studies have confirmed significant improvements in left ventricular systolic function and efficacy in preventing heart failure in subsequent pregnancies in women with PPCM who were treated with bromocriptine, the decision to prescribe bromocriptine should be made on a case-by-case basis.

### 4.2. Clinical Interpretation

In 50% to 80% of cases, recovery of normal left ventricular systolic function above 50% occurs within the first 6 months after the onset of symptoms. Lack of improvement after 6 or 12 months indicates irreversible myocardial damage in most cases. Normal right ventricular function, an LVEF greater than 30%, and an LVEDD less than 6.0 cm at the time of the peripartum cardiomyopathy diagnosis are independent prognostic factors associated with a greater chance of recovery.

### 4.3. Potential Therapeutic Targets

In recent years, we have observed significant changes in the way cardiovascular diseases are diagnosed and treated. New guidelines and innovative therapies are emerging, and increasing attention is being paid to individualizing therapeutic decisions. All of this requires us, as physicians, not only to update our knowledge but also to apply it practically in everyday clinical practice, in our daily work with patients. It is essential to focus on the practical aspects of implementing new recommendations. Despite advances in understanding the pathophysiology of PPCM, effective preventive and innovative treatment strategies remain limited, particularly for pregnant women and during the postpartum period.

### 4.4. Review Limitations

PPCM is a relatively rare condition, and the number of patients in clinical or randomized controlled trials is small. These underpowered studies do not allow for strong, statistically and clinically significant conclusions and recommendations.

Comparisons of outcomes between these different studies or subsets with or without the addition of bromocriptine to standard heart-failure therapy in patients with PPCM are limited. It seems that giving up breastfeeding and starting bromocriptine therapy in patients with PPCM should be based on a rigorous, large randomized controlled study comparing the use of bromocriptine versus placebo in addition to standard heart failure therapies in patients with PPCM.

### 4.5. Research Interpretation and Future Directions

Future research should focus on clarifying the molecular mechanisms of peripartum cardiomyopathy. Prospective studies in larger, multicenter cohorts are necessary to develop targeted therapies that could improve outcomes for both mother and child.

## 5. Conclusions

Early diagnosis and treatment of PPCM are beneficial prognostic factors, yet reliable predictive biomarkers remain lacking. Prolactin fragments are difficult to measure, and while sFlt-1 levels do correlate with the class of heart failure and the risk of complications, they do so with low specificity. The management of PPCM is similar to the standard treatment for heart failure with a reduced left ventricular ejection fraction (HFrEF), in accordance with the guidelines and taking into account the contraindications of certain medications during pregnancy. It seems that in addition to standard heart failure therapy and anticoagulation, bromocriptine should be considered. Extracorporeal membrane oxygenation (ECMO), extracorporeal membrane resuscitation (ECPR), and left ventricular assist devices (LVADs) are valuable modern forms of cardiac and circulatory support. These are used in patients with severe PPCM as a bridge to heart transplantation or, less frequently, as a destination therapy. Although modern medications such as ARNI and SGLT2 have demonstrated clinical benefits in the treatment of heart failure with reduced ejection fraction (HFrEF), their use in PPCM requires special caution, and evidence supporting their usefulness in PPCM remains limited. Therefore, their use in PPCM is considered off-label.

Baseline left ventricular end-diastolic diameter (LVEDD) and left ventricular ejection fraction (LVEF) are the main prognostic factors. LVEF less than 30%, significant left ventricular dilatation, LVEDD ≥ 6.0 cm, and right ventricular involvement are factors indicative of a poor prognosis.

## Figures and Tables

**Table 1:** The most important conclusions from the available literature data are described below and summarized in Table 1.

Study and Year of Publication	Experimental Methods	The Aim of the Study	The Characteristics of the Studied Groups and Results	Key Findings
Hilfiker-Kleiner et al. 2017 [57]	A multicenter randomized study	This randomized multicenter trial aimed to compare the effects of prolonged bromocriptine treatment vs. short-term treatment sufficient to stop lactation in addition to guideline-based heart failure therapy on LV function and clinical outcomes in patients with PPCM.	63 PPCM patients with LVEF ≤35% were randomly assigned to short-term (bromocriptine, 2.5 mg, 7 days) or long-term bromocriptine treatment (5 mg for 2 weeks followed by 2.5 mg for 6 weeks) in addition to standard heart failure therapy. *Results*: LVEF increased from 28 ± 10% to 49 ± 12% in the short-term and from 27 ± 10% to 51 ± 10% in the long-term bromocriptine treatment group. Full recovery (LVEF ≥ 50%) was observed in 52% of the short-term and in 68% of the long-term bromocriptine treatment group. No differences in hospitalization for heart failure between the two groups. The risk within the long-term bromocriptine treatment group to fail full recovery after 6 months tended to be lower. No patient in the study needed heart transplantation, LV assist device, or died.	The addition of bromocriptine to standard heart failure therapy in PPCM patients was associated with a high rate of full LV-recovery and low morbidity and mortality in PPCM patients: A better full recovery in the long-term bromocriptine treatment group was suggested.
Sliva et al., 2010 [58]	A prospective, single-center, randomized, open-label, proof-of-concept pilot study.	To determine whether bromocriptine may have beneficial effects in women with an acute onset of PPCM.	10 women with newly diagnosed PPCM receiving standard therapy and 10 women with standard therapy of PPCM plus bromocriptine for 8 weeks were included in this study. There were no significant differences in baseline characteristics, including serum 16-kDa prolactin levels and cathepsin D activity, between the 2 study groups. *Results*: Patients with bromocriptine displayed greater recovery of left ventricular ejection fraction (27% to 58%; *p* = 0.012) compared with PPCM patients and standard therapy without bromocriptine patients (27% to 36%) at 6 months. One patient in the PPCM standard therapy with the addition of bromocriptine group died compared with 4 patients in the PPCM standard therapy without the bromocriptine group. Significantly fewer PPCM with bromocriptine treatment experienced the poor outcome defined as death, New York Heart Association functional class III/IV, or LVEF <35% at 6 months compared with the PPCM patients in the group without bromocriptine treatment. Cardiac magnetic resonance imaging revealed no intracavitary thrombi. Infants of mothers in both groups showed normal growth and survival.	The addition of bromocriptine to standard heart failure therapy appeared to improve LVEF and a composite clinical outcome in women with acute severe PPCM, although the number of patients studied was small and the results cannot be considered definitive.
Koczo A et al. 2019 [56]	A multicenter prospective cohort study.	Investigation of the impact of breastfeeding and prolactin on cellular immunity and myocardial recovery in the prospective, multicenter Investigations in Pregnancy Associated Cardiomyopathy (IPAC) study.	100 women with newly diagnosed PPCM with LVEF ≤45% and recent-onset nonischemic cardiomyopathy presenting in late pregnancy or early postpartum without evidence of pre-existing structural heart disease were enrolled within the first 13 weeks postpartum at 30 centers. *Results*: The authors found no evidence that prolactin influenced myocardial recovery, and that enhancing prolactin levels by continuing to breastfeed has any adverse impact on subsequent LVEF. But they did not address whether an increase in the 16-kDa fragment might be associated with poorer myocardial recovery. The subset of women who breastfed was small (15%), and these patients represented a healthier subset with a trend toward a higher LVEF and lower New York Heart Association functional class. Furthermore, this healthier subset had been expected to do better than the more acutely ill subset that either could not, or chose not, to breastfeed. Comparisons of outcomes between these different subsets was limited.	Breastfeeding does not seem to affect outcomes, which argues against the inflammatory hypothesis and argues against a significant role for prolactin as a mediator and bromocriptine as a therapy. They found no evidence to support a recommendation against breastfeeding. Women with PPCM who are more gravely ill at the time of diagnosis may potentially benefit from the prohibition of breastfeeding and bromocriptine therapy, but a recommendation regarding the use of bromocriptine in these patients with PPCM should be based on a rigorous, large randomized controlled study comparing the use of bromocriptine versus placebo in addition to standard heart failure therapies in patients with PPCM who are at higher risk for poor outcomes.
Haghikia et al., 2019 [59]	Prospective, randomized, controlled trial, conducted in 12 participating centers in Germany	To determine the therapeutic potential of bromocriptine in PPCM patients with RV involvement.	The authors examined the effect of short-term (bromocriptine, 2.5 mg, 7 days, *n* = 10) compared with long-term bromocriptine treatment (5 mg for 2 weeks followed by 2.5 mg for another 6 weeks, *n* = 14) in addition to guideline-based heart failure therapy in patients with an initial RVEF <45%. *Results*: Reduced RVEF at initial presentation was associated with a lower rate of full cardiac recovery at 6-month follow-up. Full LV recovery was present in 50% of the short-term bromocriptine treatment group and in 64% of the long-term bromocriptine treatment group (*p* = 0.678). Full RV recovery was observed in 40% of patients in the short-term and 79% in the long-term in the bromocriptine treatment group (*p* = 0.092).	Despite an overall worse outcome in patients with RV dysfunction at baseline, bromocriptine treatment in PPCM patients with RV involvement was associated with a high rate of full RV and LV recovery, although no significant differences were observed between the short-term and long-term bromocriptine treatment regimes. These findings suggest that bromocriptine, in addition to standard heart failure therapy, may also be effective in PPCM patients with biventricular impairment.
Hoevelmann et al. 2023 [60]	A single-center, prospective clinical trial.	The study aimed to systematically characterize the burden of arrhythmias occurring in patients with newly diagnosed PPCM.	Twenty-five consecutive women with PPCM were included in this single-center, prospective clinical trial and randomized to receive either 24 h-Holter ECG monitoring followed by implantable loop recorder implantation or 24 h-Holter ECG monitoring alone. *Results*: LR + 24 h-Holter monitoring yielded a higher rate of arrhythmic events than 24-h Holter monitoring alone (40% vs. 6.7%, *p* = 0.041). Non-sustained ventricular tachycardia (NSVT) occurred in four patients (16%). There were 3 patients detected by 24 h-Holter, and multiple episodes detected by ILR in one patient. One patient deceased from third-degree AV block with an escape rhythm that failed. All arrhythmic events occurred in patients with severely impaired LV systolic function. A high prevalence of potentially life-threatening arrhythmic events in patients with newly diagnosed PPCM was observed. These included both brady- and tachyarrhythmias. The importance of extended electrocardiographic monitoring is emphasized, especially in PPCM women with severely impaired LV systolic function.	ILR in addition to 24 h-Holter monitoring had a higher yield of VAs compared to 24 h-Holter monitoring alone.

## Data Availability

Data supporting reported results can be found author.
